# Comet assay to measure DNA repair: approach and applications

**DOI:** 10.3389/fgene.2014.00288

**Published:** 2014-08-25

**Authors:** Amaya Azqueta, Jana Slyskova, Sabine A. S. Langie, Isabel O’Neill Gaivão, Andrew Collins

**Affiliations:** ^1^Department of Pharmacology and Toxicology, Faculty of Pharmacy, University of NavarraPamplona, Spain; ^2^Department of Molecular Biology of Cancer, Institute of Experimental Medicine, Academy of Science of the Czech RepublicPrague, Czech Republic; ^3^Environmental Risk and Health Unit, Flemish Institute of Technological ResearchMol, Belgium; ^4^Department of Genetics and Biotechnology, Animal and Veterinary Research Centre, University of Trás-os-Montes and Alto DouroVila Real, Portugal; ^5^Department of Nutrition, University of OsloOslo, Norway

**Keywords:** DNA repair, animal studies, human biomonitoring, occupational studies, clinical studies, base excision repair (BER), nucleotide excision repair (NER), comet assay

## Abstract

Cellular repair enzymes remove virtually all DNA damage before it is fixed; repair therefore plays a crucial role in preventing cancer. Repair studied at the level of transcription correlates poorly with enzyme activity, and so assays of phenotype are needed. In a biochemical approach, substrate nucleoids containing specific DNA lesions are incubated with cell extract; repair enzymes in the extract induce breaks at damage sites; and the breaks are measured with the comet assay. The nature of the substrate lesions defines the repair pathway to be studied. This *in vitro* DNA repair assay has been modified for use in animal tissues, specifically to study the effects of aging and nutritional intervention on repair. Recently, the assay was applied to different strains of *Drosophila melanogaster* proficient and deficient in DNA repair. Most applications of the repair assay have been in human biomonitoring. Individual DNA repair activity may be a marker of cancer susceptibility; alternatively, high repair activity may result from induction of repair enzymes by exposure to DNA-damaging agents. Studies to date have examined effects of environment, nutrition, lifestyle, and occupation, in addition to clinical investigations.

## THE IMPORTANCE OF MEASURING DNA REPAIR

DNA is a molecule prone to damage from exogenous and endogenous sources with important consequences for mutagenic and carcinogenic processes. Cells possess repair systems that amend virtually all the damage before genome change can occur; repair mechanisms therefore play a crucial role in prevention of cancer. Different pathways, involving numerous groups of repair enzymes, deal with the various types of DNA damage (Friedberg et al., 2006, **Table 1**): insertion of one or a few bases followed by ligation deals with single-strand breaks (SSBs) in the sugar–phosphate backbone; homologous recombination and non-homologous end-joining deal with the more serious double-strand breaks (DSBs) in the sugar–phosphate backbone; base excision repair (BER) deals with small base alteration such as alkylation or oxidation; nucleotide excision repair (NER), the most complex repair pathway, deals with bulky adducts of different molecules covalently linked to bases, covalent bonds between adjacent bases in the same strand (intra-strand cross links), DNA–protein cross links, as well as covalent bonds across the double helix (inter-strand cross links); and finally mismatch repair deals with wrongly paired bases. All of these pathways are likely to be regulated in a different way. For instance, enzymes playing roles in BER are assumed to be constitutive since they deal with the oxidized bases produced as a result of the inevitable presence of reactive oxygen species (a by-product of respiration) while enzymes involved in NER are more likely to be inducible since they deal with lesions that are caused sporadically by exogenous agents (e.g., food mutagens, UV light).

**Table 1:** Overview of human DNA repair systems.

Repair pathway	Damage repaired	Sources of damage
Direct reversal	Alkylated base *O*^6^-methyl-G; pyrimidine dimers (by photolyase)	Alkylating agents, nitrosourease, streptozotocin, UV(C) light
Base excision repair	Oxidized bases, alkylated bases, abasic/apurinic/apyrimidinic sites, single-strand breaks	Reactive oxygen species (ROS), alkylating agents, ionizing radiation, spontaneous hydrolysis
Nucleotide excision repair	Bulky helix-distorting lesions, intra-strand cross links, DNA–protein cross links, inter-strand cross links	UV(C) light, cigarette smoke, dietary factors [aflatoxin, PAHs (benzo[a]pyrene)]
Mismatch repair	Mismatched base pairs, small insertion loops	Replication errors, minor base modifications (oxidation, alkylation)
Double-strand break repair; i.e., homologous recombination and non-homologous end-joining	Double-strand breaks	Ionizing radiation, replication errors

*Adapted from Tyson and Mathers (2007).*

DNA repair activity or potential is regarded as a valuable marker of susceptibility to mutation and cancer. Frequently, it is determined at the level of transcription by using DNA microarray techniques or by RT-PCR for selected genes involved in the different repair pathways. However, it is well known that the activity of an enzyme does not just depend on the rate of transcription and translation, and not even on the amount of protein present. Indeed, BER gene expression has been shown not to correlate with enzyme activity (Paz-Elizur et al., 2007), and so a phenotype assay seems to be more relevant. The comet assay has been widely used for measuring the repair activity of cells, and in the past decade also of tissues.

## THE ALKALINE COMET ASSAY TO MEASURE DNA REPAIR

The alkaline comet assay, in its standard version, detects DNA strand breaks (SBs) and alkali-labile sites (ALS). This technique is based on the electrophoresis of single nucleoids (DNA attached to the nuclear matrix after cell lysis and stripping of histones), giving a comet-like image with the intensity of the tail depending on the frequency of breaks which relax supercoiling and allow migration of the DNA loops containing the breaks (Cook et al., 1976; Azqueta and Collins, 2013). If nucleoids are digested with lesion-specific endonucleases, different DNA lesions can be detected: formamidopyrimidine DNA glycosylase (FPG) detects oxidized purines, formamidopyrimidines (ring-opened adenine or guanine) and ring-opened N7 guanine adducts produced by alkylating agents; 8-oxo-guanine (8-oxoG) DNA glycosylase (OGG1) detects oxidized purines and formamidopyrimidines; endonuclease III detects oxidized pyrimidines; T4 endonuclease V detects dimerized pyrimidines (induced by UV); 3-methyladenine DNA glycosylase II (AlkA) detects 3-methyladenine; finally uracil DNA glycosylase (UDG) detects uracil misincorporated in DNA (Azqueta and Collins, 2013). Several enzymes are still under consideration to be combined with the comet assay to detect other DNA lesions.

This assay was used to measure not only DNA lesions but also DNA repair from its very beginning. The first work that refers to this technique was published by Ostling and Johanson (1984) and they studied SBs rejoining in γ-irradiated L5178Y-S cells (a murine lymphoma cell line). They used the neutral version of the comet assay where DNA is not denatured. A few years later, Singh et al. (1988) published the first work using the alkaline comet assay, where the DNA helix is unwound by alkaline treatment, and as a consequence of which ALS are converted to breaks. They also used it to study SBs rejoining in X-irradiated human lymphocytes.

These two papers studied the kinetics of repair by performing the comet assay on DNA-damaged cells at different times after incubation, in what has been called the cellular repair assay or the challenge assay (Au et al., 2010; Collins and Azqueta, 2012). The standard comet assay is used to monitor the capacity of cells to rejoin breaks; but if the aforementioned lesion-specific endonucleases are used, the removal of a particular type of lesions can be assessed. It is important that the induced lesions are as “clean” as possible to give confidence that we are monitoring the repair of a specific lesion. SSBs are easily induced by a brief treatment with H_2_O_2_ or by irradiation with X- or γ-rays; oxidized purines, mainly 8-oxoG, are induced by treating the cells with the photosensitiser Ro 19-8022 plus visible light; alkylated bases are produced by treating the cells with an alkylating agent such as methyl methanesulfonate (MMS) and dimerized pyrimidines are produced by irradiating the cells with UV(C). An optimal dose of irradiation or concentration of chemicals should be used, avoiding saturation of the DNA repair capacity of the cells or the assay. Rejoining of SSBs is a simple process that can go to completion in less than half an hour, while the repair of DSBs or oxidized bases can take hours; thus precise monitoring is required, with several measurements at suitable intervals (rather than a single measurement of damage remaining at one time point) and the repair capacity expressed as *t*_1/2_ for removal of damage or initial slope of the curve (Collins and Azqueta, 2012). A different modification of the cellular repair assay is needed to study cross link repair, since in this case the movement of DNA during electrophoresis is blocked by the cross links. Therefore, at each incubation time-point, cells are treated with an agent such as X-rays to induce breaks before performing the comet assay; repair is indicated by an increase in comet tail intensity as the blockage of X-ray-induced migration is progressively released (Spanswick et al., 2010).

A modified version of the challenge assay, the Comet-FISH assay – a combination of the comet assay with fluorescent *in situ* hybridization (FISH), using labeled probes to particular DNA sequences – has been used to study DNA repair of single genes or DNA sequences (Shaposhnikov et al., 2011). In this assay, the DNA damage repair in a specific gene can be monitored by following the “retreat” of the gene-specific signals from the comet tail to the comet head over time. In addition, the Comet-FISH assay can be used as an alternative to Southern-blotting and ligation-mediated PCR techniques to study transcription-coupled repair (TCR) of physiologically relevant levels of DNA lesions (Spivak et al., 2009; Guo et al., 2013).

Another approach to measuring the DNA repair activity with the comet assay is to measure the accumulation of DNA breaks, as incision events, by blocking repair synthesis. This approach has been used to measure NER, employing inhibitors (aphidicolin, or cytosine arabinoside in combination with hydroxyurea) of the DNA polymerase that participates in this repair pathway (Gedik et al., 1992; Vande Loock et al., 2010).

## THE COMET-BASED “*IN VITRO*” DNA REPAIR ASSAY

**FIGURE 1:**
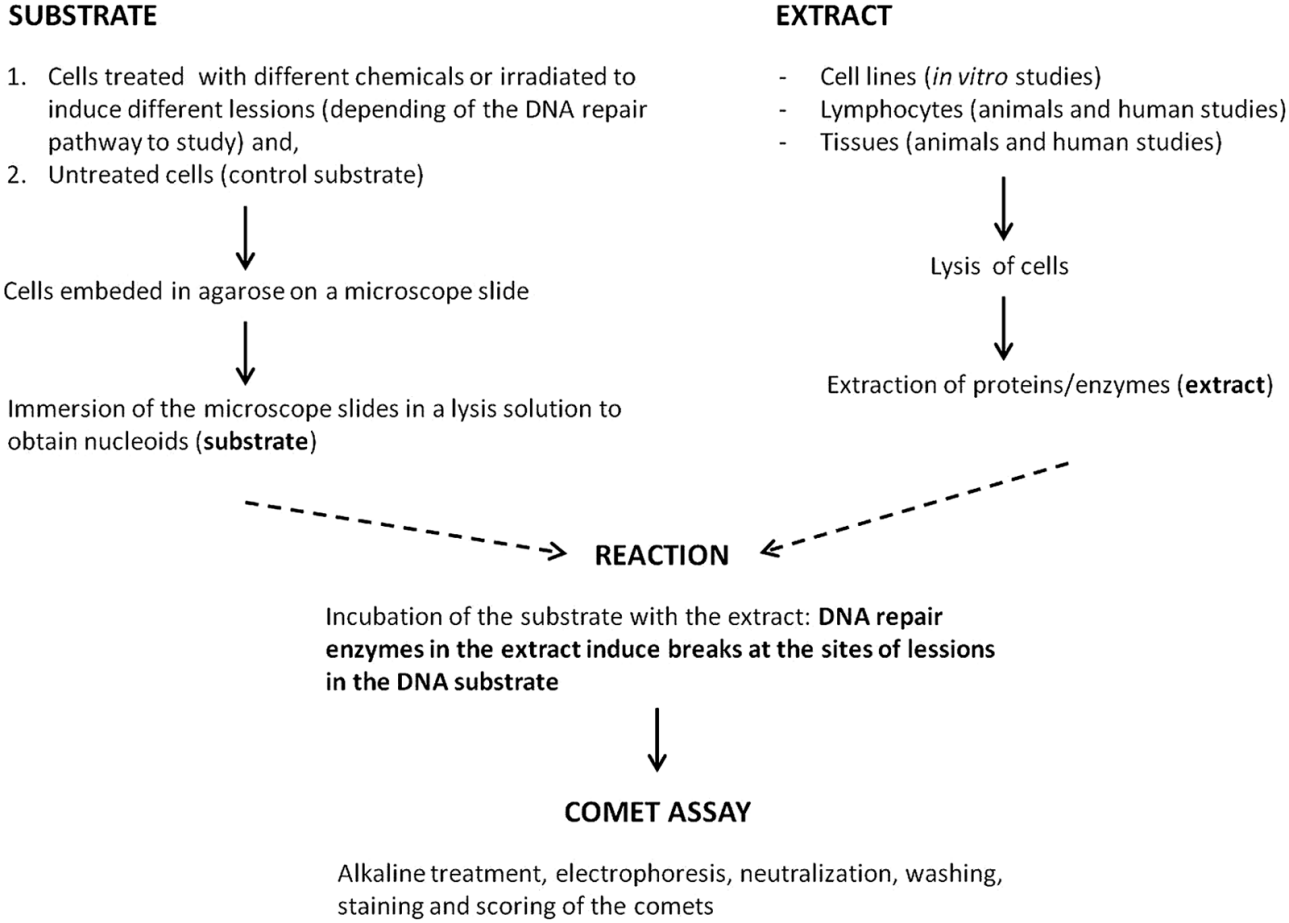
**Scheme of the comet-based *in vitro* DNA repair assay**.

The above approaches to measure DNA repair activity are not ideal for biomonitoring trials where many samples have to be processed at the same time. For this scene another strategy to measure BER or NER in cells using the alkaline comet assay has been developed (Collins et al., 2001; Langie et al., 2006; Gaivão et al., 2009; van Dyk et al., 2010; Hasplova et al., 2012). It is a biochemical approach, called the comet-based *in vitro* assay, in which DNA nucleoids containing a specific lesion (the substrate; derived by lysis of cells that have been treated with an appropriate damaging agent) are incubated with a cell extract containing a certain amount of repair enzymes (**Figure 1**). These enzymes, as a part of the repair process, induce breaks at the site of the lesions in the substrate that are measured using the alkaline comet assay protocol. The capacity of the cell extract to carry out the incision, considered to be the rate-limiting step of the repair process, is taken as an indicator of the DNA repair activity of those cells. Collins et al. (1994) demonstrated, using an early version of this assay, that the extract is capable of finishing the NER process if deoxyribonucleotides and ATP are provided. The nature of the lesions in the substrate nucleoids defines the repair pathway that it is going to be studied; for example BER can be measured with nucleoids containing 8-oxoG (induced by the photosensitizer Ro 19-8022 plus light) and NER with nucleoids containing dimerized pyrimidines [induced by UV(C)]. Substrate nucleoids should contain an excess of lesions for the extract to work, but unwanted lesions, including breaks, should be low. The time of incubation of the extract with the substrate should also be critically chosen to be able to differentiate levels of repair activity between extracts. It is also crucial to include in a parallel incubation non-damaged substrate nucleoids to determine the action of non-specific nucleases (Azqueta et al., 2009; Gorniak et al., 2013).

The current review will give an overview of the various studies in which the comet-based *in vitro* DNA repair assay has been applied so far, highlighting the most important findings as well as discussing shortcomings. The focus will not be on the practical challenges that might arise when applying the assays, since the sources of potential problems and practical advices have been published recently (Azqueta et al., 2013a; Slyskova et al., 2014c) together with a detailed protocol of this approach to measure BER and NER in cultured cell lines, blood cells, animal tissues, and human biopsies. A comet-based *in vitro* assay for cross link repair has also been developed (Herrera et al., 2009).

## STUDIES USING THE COMET-BASED *IN VITRO* DNA REPAIR ASSAY

The comet-based *in vitro* DNA repair assay has been used in some cell culture and animal studies but it is mostly used in human biomonitoring. In this section, we will briefly review the different *in vitro*, *in vivo* animal and human studies where this technique has been applied to measure DNA repair activity.

### CELL CULTURE STUDIES

There are very few studies in the literature where the comet-based *in vitro* DNA repair assay has been applied. Silva et al. (2008) published the first paper using this technique to measure BER activity in cell culture. They studied the effect of different polyphenols on the BER activity of PC12 cell (derived from rat pheochromocytoma) and found a significant increase in the incision activity of extracts from cells treated with rosmarinic acid. A year later they examined two synthetic nitrogen compounds, developed as antioxidant drugs, but they did not find any such effect on repair (Silva et al., 2009). Also Sliwinski et al. (2008) published the first paper using the technique to measure NER activity in cell culture. They measured the effect of ST1571, a drug used in the treatment of chronic myeloid leukemia which inhibits the activity of the BCR/ABL oncogenic kinase, on the NER activity of different human lymphoid leukemia cells. They found that extract from BCR/ABL cells treated with the drug showed a highly significant decrease in incision activity.

Extract from HeLa cells (derived from human cervical cancer) and Caco-2 cells (derived from human colon carcinoma) treated with β-cryptoxanthin showed a significant increase in BER activity compared with non-treated cells (Lorenzo et al., 2009). Incubation of Caco-2 cells with water extracts of Salvia species, luteonil-7-glucoside and rosmarinic acid also increased the BER activity of the cells though it was non-significant for rosmarinic acid (Ramos et al., 2010a). The same group demonstrated a significant increase in the BER activity of extract from Caco-2 cells incubated with ursolic acid but not with luteolin (Ramos et al., 2010b).

Azqueta et al. (2013b) showed that vitamin C caused DNA breaks in nucleoids (substrate) when trying to carry out the comet-based *in vitro* repair assay to study the effect of vitamin C on BER of Caco-2 cells. This finding made it impossible to carry out this test since vitamin C was present in cell extracts and masked the results (Azqueta et al., 2009, 2013b).

The effect of hereditary tyrosinemia type 1 metabolites on DNA repair was studied by van Dyk et al. (2010) in HepG2 cells (derived from human hepatoma). This disorder, caused by a defective fumarylacetoacetate hydrolase enzyme, causes the accumulation of metabolites such as succinylacetone and *p*-hydroxyphenylpyruvate. The authors studied the BER and NER incision activity in extract from cells treated with both metabolites. They used H_2_O_2_- and methyl methanesulfonate (MMS)-treated cells to produce the nucleoids for studying BER and benzo[a]pyrene-treated cells to produce the nucleoids to study NER. Both metabolites decreased the DNA repair activity of the cells, the effects being more pronounced in BER than in NER.

In some of these studies, there is a lack of proper controls for the correct interpretation of the results. Azqueta et al. (2009) warned that non-treated nucleoids, as substrate, should always be used to allow for the possible presence of non-specific nucleases. They also pointed out the possibility that the test compound might itself directly induce breaks in the nucleoids (substrate) and its presence in the extract thus interfere with the assay.

### ANIMAL STUDIES

Although comet-based assays are easy to use, sensitive, versatile, and relatively inexpensive, to the best of our knowledge, there are only a few reports that describe the use of animal tissue extracts in the comet-based assay to measure activities of NER (Langie et al., 2010a) or BER (Mikkelsen et al., 2009; Langie et al., 2011, 2013, 2014; Gorniak et al., 2013) *in vitro*.

Mikkelsen et al. (2009) were the first to apply the *in vitro* repair assay to study BER-related DNA incision activity of protein extracts from lung and liver of aging mice. However, they did not include a control of low-damage nucleoids (e.g., for BER; nucleoids not exposed to the photosensitizer Ro 19-8022 plus light), incubated with protein extract in their assay. Inclusion of these controls is important to detect the possible presence of non-specific nuclease activity, preventing misinterpretation of the findings as has been reported by Langie et al. (2011) and Gorniak et al. (2013) for tissues and by Azqueta et al. (2009) for cultured cells. Moreover, the non-specific nuclease activity can differ markedly between various tissues in the same animal, and so direct comparisons of DNA incision activity in different tissues should be interpreted with caution. Recently, Langie et al. (2011) optimized the comet-based assay for measuring BER-related DNA incision activity in animal tissues, specifically with mouse tissues (Gorniak et al., 2013). The problem of non-specific nuclease activity was overcome by the addition of 1.5 μM aphidicolin in DMSO and selection of a reliable protein concentration, allowing specific detection of DNA repair incision activity. Whether aphidicolin could possibly enhance detection of NER activity by preventing the occurrence of non-specific nuclease activity or any repair synthesis, has not been rigorously tested yet.

So far, the comet-based *in vitro* DNA repair assay has mainly been used to study the effect of aging or nutritional interventions in animal tissues. Our recent studies (Langie et al., 2011; Gorniak et al., 2013) showed significant age-related declines in BER-related DNA incision activity in brain, lung, and colon tissues of rodents, while incision activity was observed to increase with age for liver (Mikkelsen et al., 2009; Langie et al., 2011). In addition, differences in BER-related DNA incision activity were observed between proliferative and non-proliferative tissues (Mikkelsen et al., 2009; Gorniak et al., 2013). Furthermore, dietary restriction has been shown to influence DNA repair, increasing BER activity in liver as compared to *ad libitum* fed animals (Langie et al., 2011). Recently, much effort has gone into studying the effect of prenatal dietary interventions. Langie et al. (2010a, 2014) observed maternal supplementation with micronutrients to enhance NER activity in the colon and BER activity in the hippocampus of piglet offspring. A maternal low-folate diet during pregnancy and lactation was reported to enhance BER-related incision activity in weaning mice but to reduce BER activity once the offspring reached adulthood (Langie et al., 2013).

Although measuring DNA repair in mammalian tissues using the comet-based assay remains a challenge because of the high levels of non-specific activity, the adapted and optimized assay for quantification of BER-associated incision activity in rodent tissues opens opportunities for a wide range of *in vivo* studies on BER including effects of environmental exposures (such as toxins, dietary factors and pharmaceutical agents) and of physiological processes including growth, development, degenerative diseases, and aging.

*Drosophila melanogaster* is a model organism with practical and theoretical advantages such as its ease of manipulation, its short life cycle, its xenobiotic metabolizing system (Hallström et al., 1984; Søndergaard, 1993), antioxidant enzymes, and DNA repair pathways (Sekelsky et al., 2000) that are similar or equivalent to those in mammals, and the detailed knowledge of its genome (Adams et al., 2000). It is an established insect model for human diseases and toxicological research, recommended by the European Centre for the Validation of Alternative Methods (ECVAM). Moreover, strains are available that are efficient and deficient for the several repair systems. The comet assay has been successfully applied to *Drosophila* to study not only genotoxicity but also DNA repair.

Very recently, the comet-based *in vitro* repair assay has been applied to *D. melanogaster* to measure the DNA repair activity in extracts from different strains, proficient and deficient in DNA repair, using wild-type neuroblast cells treated *in vivo* with 1 mM MMS as substrate (Gaivão et al., 2014; Rodríguez et al., submitted). This last work demonstrates the feasibility of an *in vitro* approach to *Drosophila* repair, and – by analyzing extracts of different *Drosophila* strains (such as *mus201*, *mus308* and *mus20, mus308*) – shows that genetic differences are reflected in phenotype and can be quantitated. The *in vitro* approach can provide information about the genetic basis and regulation of specific repair enzymes (Rodríguez et al., submitted).

### HUMAN STUDIES

Individual DNA repair activity is a valuable biomarker since it has been regarded as a marker of susceptibility to mutation and cancer. A high repair activity is related to a decrease of the chance of unrepaired damage when cells replicate and so to a decrease in potential mutations. On the other hand, a high repair activity can also reflect exposure to DNA-damaging agents which might induce synthesis of the repair enzymes. Anyway a high repair activity is always a good thing but more evidence is needed to confirm that the DNA repair activity is a biomarker of susceptibility to cancer.

The *in vitro* repair assay based on the comet assay has been particularly useful in human trials; samples of cells or tissue, or cell extracts, can be frozen at -80°C for long periods before the repair assay is carried out, which is advantageous when, typically, samples are collected from several subjects on the same occasion, and often other samples have to be collected and other assays performed.

Two studies have applied the *in vitro* assay in order to investigate DNA repair activity against the background of other biomarkers of genotoxicity. Etemadi et al. (2013) aimed to explain variability in PAH-related adducts among non-smokers by evaluating genetic polymorphisms and individual NER activity. In this study, phase I SNPs and NER activity explained 17% of the variation in PAH DNA-adduct levels. The association between oxidative DNA damage, antioxidant serum capacity and BER activity was investigated in healthy non-smokers, but no strong relationships were observed (Tsai et al., 2013).

Though the DNA repair activity is determined genetically, it is also affected by environmental conditions such as nutritional and lifestyle factors. As already pointed out, regulation of DNA repair activity is not simply at the level of transcription, and gene expression is not a reliable guide to enzyme activity, so there is a need for a phenotypic assay. The comet-based *in vitro* DNA repair assay has been used mainly in nutritional intervention studies but also in occupational and clinical studies, as described in the next subsections.

#### Occupational studies

Every day, human populations are exposed to mutagenic and carcinogenic compounds, both occupational and environmental. In terms of occupational exposure, in several jobs people are exposed to genotoxic/mutagenic compounds, for example: pesticides, hair dyes, formaldehyde, antineoplastic agents, organic solvents, etc.

Dusinska et al. (2004a) measured BER capacity in workers exposed to asbestos, who had significantly higher level of chromosomal aberrations than unexposed factory controls, but no effect of exposure on BER capacity was observed. In another study of the same group, BER capacity was again unaffected by exposure to mineral fibers as measured in workers of rockwool manufacture and compared with administrative employees of the same factory (Dusinska et al., 2004b). Slyskova et al. (2007) measured BER capacity by *in vitro* comet-based assay in styrene-exposed workers as compared to unexposed clerks. Base excision repair capacity did not differ between groups and did not correlate with parameters of styrene exposure or biomarkers of genotoxic effects, namely DNA strand breaks, N1-styrene-adenine DNA adducts, chromosomal aberrations and *HPRT* mutations.

In these studies, while the harmful effect of exposure was clearly recognizable by high levels of various biomarkers of genotoxicity, the effect of exposure on DNA repair activity was not that straightforward or substantial that it could have been observed in relatively small study groups, which is usually the case for occupational studies limited by the number of employees in the factory.

#### Nutritional studies

Until recently, there was little interest in the regulation of DNA repair by nutritional factors. It was generally assumed that DNA repair is a constitutive, or “housekeeping” function, unlikely to be much affected by exogenous factors. The inter-individual range of repair capacities (both BER and NER) is considerably more than can be explained by differences in genotype; polymorphisms in repair genes have been shown to have little effect on the corresponding enzyme activities. Induction of repair by exposure to DNA-damaging agents is a feasible source of variation, and several researchers have been looking also at the possibility that nutrition plays a role.

The assay was first applied to humans in a trial of coenzyme Q10 in six subjects (Tomasetti et al., 2001). After a week of supplementation with 100 mg Q10 per day, BER activity was almost three times as high as the activity before supplementation. In a crossover designed trial of green kiwifruit (one, two or three per day for 3 weeks, with washout periods between doses), there were highly significant increases in BER activity – without a clear dosage effect (Collins et al., 2003). A later study with golden kiwifruit failed to show any effect on BER or NER (using a substrate of nucleoids from UV(C)-treated cells; Brevik et al., 2011a). An increase in BER was reported with slow-release vitamin C capsules in a 4-week placebo-controlled trial (Guarnieri et al., 2008). There was no significant effect of intervention with a mix of selenium, retinol, β-carotene, vitamin C, and vitamin E for 6 weeks (Caple et al., 2010), nor after a broccoli-rich diet for 10 days in a crossover trial (Riso et al., 2010). A diet rich in fruits and vegetables (600 g/day) resulted in no effect on BER (Guarnieri et al., 2008), whereas a similar study of the effects of antioxidant-rich fruits and vegetables (Brevik et al., 2011b) showed a significant increase in BER (and a smaller, non-significant increase with three green kiwifruits/day). In the latter study, NER activity was also studied; in this case, repair activity was decreased by both the fruit and vegetable diet and the addition of kiwifruits to the normal diet. This unexpected finding gave rise to the speculation that a lower level of DNA damage resulting from primary protection by phytochemicals led to a failure to induce secondary protection in the form of NER enzymes; in other words, repair activity was not needed as the damage level was low. The NER assay was applied by Langie et al. (2010b) using a substrate containing benzo(a)pyrene diol epoxide-induced bulky adducts; enhanced repair was seen only in subjects carrying multiple low-activity alleles of repair genes. Recently, Slyskova et al. (2014a) analyzed BER and NER capacities in a large cohort of 340 healthy individuals examined for antioxidants intake by food frequency questionnaires and antioxidants plasma levels. They observed that while BER was not associated with antioxidant-rich diet intake, NER was positively correlated with plasma levels of ascorbic acid and α-carotene.

In summary, while it is evident that nutritional factors can influence DNA repair phenotype, results tend to be inconsistent between studies, and further investigations are needed. At present we have no clear indication as to how the modulation of repair is effected; it seems not to be via changes in gene expression (as discussed by Collins et al., 2012).

#### Clinical studies

In studies investigating DNA repair activity in relation to human diseases, the cellular or challenge assay has commonly been applied; the *in vitro* DNA repair comet-based assay has been used only rarely, on peripheral blood cells of study subjects, but also on tumor samples.

Base excision repair has been assessed in patients suffering chronic renal failure, showing no association between BER activity and duration of hemodialysis (Stoyanova et al., 2014). Slyskova et al. (2012) determined BER and NER activities in tumors from colorectal cancer patients and observed that the activities of both pathways did not differ from those of healthy adjacent tissue. This study however showed the positive correlation of both pathways between peripheral lymphocytes and colon mucosa, supported also by Herrera et al. (2009).

Since *in vitro* DNA repair comet-based assay to study BER and NER in human solid tissues was optimized only recently (Slyskova et al., 2012, 2014b), more clinical studies on DNA repair in relation to tissue-specific diseases might be expected to be released in the near future.

## CONCLUDING REMARKS

The *in vitro* comet-based DNA repair assay is simple and versatile. Base excision repair or NER can be measured by using substrate nucleoids with appropriate DNA lesions. The assay is particularly suitable where many samples need to be assessed and compared in a short time, which is the case in human molecular epidemiology studies of occupational exposure, nutrition, lifestyle, aging, etc.

Integration and comparison of results from different laboratories is only possible if standard protocols are adopted. The assay depends critically on the protein concentration in the extract, reflecting the number of cells or amount of tissue used; this should therefore be constant. Validation of the assay against other repair assays is still needed, and a ring-study to compare assay performance in different laboratories should be carried out.

Results to date have demonstrated the range of repair activities in a healthy human population – a range far greater than can be explained by genetic polymorphisms. This emphasizes the importance of regulation of repair by environmental and/or intrinsic factors – about which we still know relatively little.

## Conflict of Interest Statement

The authors declare that the research was conducted in the absence of any commercial or financial relationships that could be construed as a potential conflict of interest.
